# Prospective Rates, Longitudinal Associations, and Factors Associated With Comorbid Insomnia Symptoms and Perceived Cognitive Impairment

**DOI:** 10.3389/fnins.2021.817933

**Published:** 2022-01-24

**Authors:** Sheila N. Garland, Hans Ivers, Josée Savard

**Affiliations:** ^1^Department of Psychology, Faculty of Science, Memorial University, St. John’s, NL, Canada; ^2^Discipline of Oncology, Faculty of Medicine, Memorial University, St. John’s, NL, Canada; ^3^School of Psychology, Université Laval, Quebec City, QC, Canada; ^4^CHU de Québec-Université Laval Research Center, Quebec City, QC, Canada; ^5^Laval University Cancer Research Center, Quebec City, QC, Canada

**Keywords:** cancer, cognition, impairment, insomnia, sleep

## Abstract

**Background:**

Insomnia and cognitive impairment are both common conditions experienced by people diagnosed with cancer. Individually, these conditions have negative impacts on functioning, but the combined burden has yet to be evaluated. The purpose of this research was to estimate rates of comorbid insomnia and perceived cognitive impairments, examine the longitudinal associations between these two conditions, and identify demographic and clinical factors associated with reporting both insomnia and perceived cognitive impairment.

**Methods:**

In this secondary analysis, a heterogeneous sample of 962 patients completed the Insomnia Severity Index (ISI) and the Cognitive Failures Questionnaire (CFQ) at the time of their cancer surgery (baseline; T1) and then again at 2 (T2), 6 (T3), 10 (T4), 14 (T5), and 18 (T6) months. Correlations and partial correlations, controlling for age and education level, were computed at each time point to assess the relationship between ISI and CFQ scores. Cross-lagged correlations assessed associations between ISI and CFQ scores over time. Proportions of patients with comorbid insomnia and cognitive impairments were calculated and logistic regressions investigated changes over time in these proportions. ANOVAs, logistic regressions, ordinal regressions, and multinomial regressions were used to identify risk factors of having comorbid insomnia and cognitive difficulties.

**Results:**

Significant and bidirectional correlations between ISI and CFQ scores were observed at each time point and over time. The proportion of patients having both clinical levels of insomnia and perceived cognitive difficulties ranged from 18.73 to 25.84% across time points and this proportion was significantly greater at T1 and T2 than T4, T5, and T6. Participants who reported comorbid insomnia and cognitive impairment were more likely to be younger, female, not currently working, currently receiving chemotherapy, with clinical levels depression and anxiety, and using antidepressants or anxiolytics.

**Conclusion:**

Comorbid insomnia and perceived cognitive impairment affects around one in five patients and is more frequent at the beginning of the cancer care trajectory. The relationship between insomnia and cognitive impairment appears to be bidirectional. Insomnia may represent an important patient level vulnerability that when identified and treated can improve perception of cognitive function.

## Introduction

Insomnia is a prevalent and persistent condition in people diagnosed with cancer throughout their treatment trajectory (Palesh et al., 2010; Leysen et al., 2019). Insomnia is defined as dissatisfaction with sleep quality or quantity characterized by difficulty initiating or maintaining sleep that causes significant distress and impairment in functioning (American Psychiatric Association, 2013). When symptoms occur at least 3 times per week and have been present for at least 3 months, the diagnostic criteria for insomnia disorder are met. Estimates suggest that roughly 40–60% of patients will present with insomnia symptoms and that around 20% will meet the criteria for a disorder at the time of diagnosis (Savard et al., 2011; Fleming et al., 2018; Harrold et al., 2020). Even after treatment completion, the prevalence of insomnia symptoms or disorder remains up to three times greater than in the general population, with a prevalence of insomnia symptoms of 47% at 12 months (Fleming et al., 2018) and 36% at 18 months (Savard et al., 2011) compared to roughly 6–10% (Ohayon, 2002; Buysse, 2013). Patients who experience insomnia at the time of their cancer diagnosis are significantly more likely to report persistent insomnia, compared to those who identify as good sleepers (Savard et al., 2011), which emphasizes the importance of early identification and intervention. Factors associated with cancer-related insomnia include younger age (Harrold et al., 2020), having a diagnosis of a breast or gynecological cancer (Savard et al., 2011; Harrold et al., 2020), receiving chemotherapy (Fleming et al., 2018), and experiencing comorbid anxiety or depression (Hoang et al., 2019; Maguire et al., 2019). Insomnia in cancer survivors has been associated with greater overall symptoms burden including higher levels of pain, fatigue, anxiety, and depression (Nishiura et al., 2015). Insomnia has also been linked to an increased risk of infections (Ruel et al., 2015), worse long-term quality of life (Lowery-Allison et al., 2018), and poorer treatment response and greater overall mortality (Innominato et al., 2015), although the latter needs to be demonstrated in more studies. As such, insomnia is an important treatment target to optimize short and long-term cancer recovery.

Cognitive impairment is also a common, but less well-understood, complaint of people diagnosed and treated for cancer. Compared to non-cancer controls, neuropsychological testing demonstrates a range of objective deficits in memory, concentration, information processing and executive functioning in some but not all cancer survivors (Ahles and Root, 2018; Janelsins et al., 2018). A systematic review of 101 cross-sectional, longitudinal, and randomized controlled studies reported more cancer-related cognitive alterations for subjective measures than performance tasks (Bray et al., 2018). In a prospective observational study, 45% of women with breast cancer 6 months post-chemotherapy (*n* = 581) reported a clinically meaningful decline in perceived cognition, compared to 12% of non-cancer controls (*n* = 334) (Janelsins et al., 2017). As with insomnia, younger age and higher levels of anxiety and depression are also associated with greater cognitive complaints. Cognitive impairment has a significant negative impact on quality of life, including return to work, social relationships, and self-confidence (Lange et al., 2019), which makes the identification of potentially modifiable risk factors a research and clinical imperative.

In the general population, insufficient quality and quantity of sleep has been found to be associated with numerous cognitive deficits including impaired psychomotor and cognitive speed, attention, learning, memory, and executive function (Goel et al., 2009). More specifically, insomnia is associated with worse working memory, attention, and perceived cognitive impairment (Brownlow et al., 2020). The cognitive effects of insomnia in cancer patients has not been well studied. An early cross-sectional study of women with breast cancer found that those with an insomnia disorder had worse verbal episodic memory and executive functioning assessed objectively (Caplette-Gingras et al., 2013). In a more recent cross sectional online survey of 1,393 breast cancer survivors, 47% showed current cognitive complaints and those who reported sleep difficulties were twice as likely to report cognitive impairments (Boscher et al., 2020). When assessed prospectively in a different sample of 98 women with breast cancer from the time of diagnosis to 12 months, rates of perceived cognitive impairments were stable at roughly 36% (Rodriguez et al., 2021). While fatigue and insomnia were associated with greater perceived cognitive impairment, insomnia did not significantly predict future cognitive impairment (Rodriguez et al., 2021). In men with prostate cancer, insomnia symptoms significantly mediated the relationship between receiving androgen deprivation therapy and perceived cognitive function and satisfaction with cognition (Garland et al., 2021b). In sum, although existing research suggests an association between cancer-related insomnia and both objective and perceived cognitive impairment, it is not yet clear the extent to which insomnia precedes or follows cognitive impairments, how commonly these symptoms co-exist and what factors may influence this co-morbidity. Further, studies have investigated only a few specific groups of cancer patients, often breast cancer, which limits the generalizability of results.

The objectives of this secondary analysis were to estimate rates of comorbid insomnia and perceived cognitive impairments and examine the longitudinal associations between these two conditions in the 18 months following a cancer surgery. Further, we sought to identify factors that are associated with reporting both insomnia and perceived cognitive impairment, compared to those individuals who only report one of the symptoms or none.

## Materials and Methods

### Participants

This is a secondary analysis of a larger study on the epidemiology of cancer-related insomnia (for details, see Savard et al., 2009, 2011). Patients were eligible if they were scheduled to undergo a surgery with a curative intent for a first non-metastatic cancer, were between 18 and 80 years old and were able to read and understand French. They were excluded if they had received a neoadjuvant treatment for cancer, the surgery was part of brachytherapy for prostate cancer, had severe cognitive impairments (e.g., Alzheimer’s disease) or a severe psychiatric disorder (e.g., psychosis, bipolar disorder), had received a diagnosis for a sleep disorder other than insomnia (including sleep apnea), and had severe visual, hearing or language defects. A total of 3,196 patients were solicited, 1,677 were eligible (52.5% of solicited patients), and 962 agreed to participate (57.4% of eligible patients).

### Procedure

Patients were approached by a research assistant to participate to the study on the day of their pre-operative visit at L’Hôtel-Dieu de Québec and the Hôpital du St-Sacrement of the CHU de Québec-Université Laval. The study was approved by the ethics review boards of both hospitals. After the study goals and procedures were explained, agreeing patients were invited to provide written consent to participate. They also received a battery of self-report scales to be completed within the next 2 weeks (baseline; T1) and received by mail the same questionnaires to fill out 2 (T2), 6 (T3), 10 (T4), 14 (T5), and 18 (T6) months later. Of note, 81.2% of the participants completed baseline measures after the surgery (20 days after on average).

### Measures

Data on cancer and treatment-related information was abstracted from patients’ medical charts. Participants self-reported other demographic variables.

**Insomnia Severity Index (ISI):** The ISI contains 7 items designed to specifically assess the severity of insomnia symptoms, their impact on daytime functioning, and the amount of associated distress in the past week (Savard et al., 2005). The ISI is summed to compute a total score with a range of 0–28. Higher values indicate more severe insomnia severity and a score of ≥ 8 indicates the presence of clinical levels of insomnia.

**Cognitive Failures Questionnaire (CFQ):** The CFQ is a 25-item measure of self-reported failures in perception, memory, and motor function in the past 6 months at T1 and since the last questionnaire completion from T2 to T6 (Broadbent et al., 1982). Responses are rated on a 5 point scale from 0 (never) to 5 (very often), yielding a total score of 0–100. The higher the score, the more cognitive impairment. The psychometric qualities of this questionnaire are satisfactory (Broadbent et al., 1982) and this measure is frequently used to assess self-reported cognition in cancer patients (Bray et al., 2018). While there is no universally agreed upon cutoff score to distinguish clinically significant levels of perceived cognitive impairment using this instrument in cancer patients, past research in cardiac patients have used scores ≥ 26 (Moulaert et al., 2017), > 32 (Boyce-van der Wal et al., 2015), and > 44 (Steinbusch et al., 2017). We decided to use a cutoff > 32, which appeared to be a good compromise between having a too liberal or a too conservative criterion.

### Statistical Analyses

Correlations and partial correlations, controlling for age and education level, were computed at each time point to assess the relationship between insomnia (ISI) and cognitive functioning (CFQ) scores. Cross-lagged correlations of ISI scores with subjective cognitive functioning (CFQ) were also computed between one variable at one time point and the other variable at the previous time point, and this, by including in the calculation all five possible combinations of a time point and the previous time point (i.e., T1-T2, T2-T3, T3-T4, T4-T5, and T5-T6). Descriptive analyses (frequencies, percentages) were conducted to estimate, at each time point, the proportion of patients who had both clinical levels of insomnia (ISI ≥ 8) and perceived cognitive impairments (CFQ > 32), who had only one condition and who had none. Logistic regressions with repeated measures, including a random effect for patients, were computed to investigate changes over time in these proportions. Finally, to identify risk factors of having comorbid insomnia and cognitive difficulties, ANOVAs (for continuous variables), logistic regressions (for dichotomous variables), ordinal regressions (for ordinal variables) and multinomial regressions (for categorical variables with more than two categories) were conducted to compare patients having both conditions to the remaining on: age, sex, education, marital status, occupation, income, cancer type, cancer stage, current and past chemotherapy, current and past radiation therapy, current hormone therapy, medical comorbidity, menopausal status (for females), presence of clinical levels of depression and anxiety, and the use of antidepressants and anxiolytics. With the exception of education and income, for which baseline data were used, all models included data at all time points and a random patient effect to take into account the dependence between data collected from the same patient throughout the study.

## Results

### Participants’ Characteristics

The mean age was 57.0 years old (*SD* = 9.9; range: 23–79) and 64.4% of the sample was composed of females. Time since cancer diagnosis was 2.2 months on average (range: 0.1–7.1). The cancer types were breast (48.3%), prostate (27.3%), gynecological (11.5%), urinary and gastro-intestinal (7.2%), and other (5.7%). Most commonly, patients had Stage 1 (35.2%) or Stage 2 (37.2%) disease. More information is available elsewhere (Savard et al., 2009, 2011).

### Associations Between Insomnia and Perceived Cognitive Impairments

Correlations obtained between ISI and CFQ scores and partial correlations controlling for age and education are presented in Table 1. Correlations were all significant (all *p*s < 0.0001) and varied between 0.25 and 0.35 across time, while partial correlations were also all significant (all *p*s < 0.0001) and ranged from 0.23 to 0.37. In both cases, the lowest correlation coefficients were found at baseline (T1).

**TABLE 1:** Correlations and partial correlations, controlling for age and education, between ISI and CFQ scores.

Time	Correlation	Partial correlation
T1 (0 month)	0.25****	0.23****
T2 (2 months)	0.35****	0.35****
T3 (6 months)	0.32****	0.32****
T4 (10 months)	0.35****	0.35****
T5 (14 months)	0.34****	0.37****
T6 (18 months)	0.33****	0.33****

*ISI, Insomnia Severity Index; CFQ, Cognitive Failures Questionnaires.*

****p < 0.0001.

Significant cross-lagged partial correlations, controlling for age and education, were obtained between ISI and CFQ scores, where ISI scores at one time point was significantly associated with CFQ scores at the previous time point (previous CFQ → current ISI: *r* = 0.30, *p* < 0.001) and CFQ scores at one assessment was significantly correlated with ISI scores at the previous time point (previous ISI → current CFQ; *r* = 0.31, *p* < 0.001).

### Mean Scores and Rates of Insomnia and Perceived Cognitive Impairments Across Time

Participants obtained mean ISI scores of 8.89 (*SD* = 6.1) at T1, 8.83 (*SD* = 5.6) at T2, 7.65 (*SD* = 5.7) at T3, 6.99 (*SD* = 5.4) at T4, 6.85 (*SD* = 5.4) at T5, and 6.47 (*SD* = 5.1) at T6. The mean CFQ scores were 29.23 (*SD* = 12.6), 29.04 (*SD* = 13.6), 29.30 (*SD* = 13.2), 29.19 (*SD* = 13.4), 28.90 (*SD* = 13.3), and 28.69 (*SD* = 13.0), respectively. Rates of clinical levels of cognitive impairments varied from 37.18 to 39.88% with little change over time, while rates of clinical insomnia ranged from 35.63 to 55.88% with the highest proportions found at T1 (see Table 2).

**TABLE 2:** Rates (n;%) of clinical levels of insomnia and perceived cognitive impairments and comorbidity rates.

	With clinical cognitive impairments	Without clinical cognitive impairments	Total with clinical insomnia
**T1 (*n* = 952)**			
With clinical insomnia	246 (25.84%)	286 (30.04%)	**532 (55.88%)**
Without clinical insomnia	120 (12.61%)	300 (31.51%)	
Total with clinical	**366 (38.45%)**		
cognitive impairments			
**T2 (*n* = 850)**			
With clinical insomnia	219 (25.76%)	247 (29.06%)	**466 (54.82%)**
Without clinical insomnia	103 (12.12%)	281 (33.06%)	
Total with clinical	**322 (37.88%)**		
cognitive impairments			
**T3 (*n* = 810)**			
With clinical insomnia	185 (22.84%)	173 (21.36%)	**358 (44.20%)**
Without clinical insomnia	138 (17.04%)	314 (38.77%)	
Total with clinical	**323 (39.88%)**		
cognitive impairments			
**T4 (*n* = 769)**			
With clinical insomnia	158 (20.55%)	140 (18.21%)	**298 (38.75%)**
Without clinical insomnia	138 (17.95%)	333 (43.30%)	
Total with clinical	**296 (38.49%)**		
cognitive impairments			
**T5 (*n* = 727)**			
With clinical insomnia	148 (20.36%)	128 (17.61%)	**276 (37.96%)**
Without clinical insomnia	135 (18.57%)	316 (43.47%)	
Total with clinical	**283 (38.93%)**		
cognitive impairments			
**T6 (*n* = 710)**			
With clinical insomnia	133 (18.73%)	120 (16.90%)	**253 (35.63%)**
Without clinical insomnia	131 (18.45%)	326 (45.92%)	
Total with clinical	**264 (37.18%)**		
cognitive impairments			

*The bold refers to the total number of participants with clinical levels of insomnia or cognitive impairment.*

### Comorbidity of Insomnia and Perceived Cognitive Impairments

The proportion of patients having both clinical levels of insomnia and perceived cognitive difficulties ranged from 18.73 to 25.84% across time points (see Table 2). Most participants had scores under the clinical cutoff for both insomnia and cognitive impairments (from 31.51 to 45.92%). Having clinical levels of cognitive impairments with no clinical levels of insomnia was the least frequent situation (from 12.12 to 18.57%). The proportion of patients having comorbid insomnia and cognitive impairments significantly changed over time, *F*(5, 1,972) = 5.20, *p* < 0.0001. Pairwise comparisons indicated that this proportion was significantly greater at T1 and T2 than T4, T5, and T6. A significant time effect was also found on rates of clinical insomnia only, *F*(5, 797.8) = 17.77, *p* < 0.0001. Pairwise comparisons revealed that rates obtained as T1 and T2 were significantly greater than those observed from T3 to T6. The proportion of patients with clinical cognitive impairments only significantly changed over time, *F*(5, 2,665) = 13.90, *p* < 0.0001, with significantly lower rates at T1 and T2 than from T3-T6. Finally, a significant time effect was shown on rates of patients with absence of clinical levels on any of the symptoms, *F*(5, 914.8) = 16.83, *p* < 0.0001. Pairwise comparisons indicated that these proportions were significantly lower from T1 to T3 than from T4 and T6 and that they were significantly lower at T1 and T2 than T3 and T5.

### Risk Factors for Comorbid Insomnia and Perceived Cognitive Impairments

Among all possible risk factors investigated, differences between patients with and without comorbid insomnia-perceived cognitive impairments were significant on age, sex, occupation, current chemotherapy, the presence of clinical levels of depression and anxiety, and the use of antidepressants and anxiolytics. More specifically, patients with comorbidity were significantly younger, *F*(1, 3,701) = 21.81, *p* < 0.0001 (57.46 vs. 57.63), more likely to be females, *F*(1, 4,816) = 7.20, *p* < 0.01 (83.32% vs. 73.28%), to be currently receiving chemotherapy *F*(1, 1,747) = 6.22, *p* < 0.05 (4.98% vs. 3.63%), and to use antidepressants, *F*(1, 4,635) = 6.90, *p* < 0.01 (8.35% vs. 5.47%) and anxiolytics, *F*(1, 4,635) = 36.30, *p* < 0.0001 (29.77% vs. 16.02%). They were also less likely to work, *F*(1, 4,801) = 7.03, *p* < 0.01 (24.22% vs. 30.58%) and to report a clinical level of depression, *F*(1, 4,804) = 7.70, *p* < 0.01 (95.64% vs. 97.35%), and anxiety, *F* (1, 4,806) = 26.00, *p* < 0.0001 (96.94% vs. 99.12%).

## Discussion

To our knowledge, this is the first study to prospectively assess the comorbidity of insomnia symptoms and perceived cognitive impairment in a large, heterogeneous sample of cancer patients. Over an 18-month period post-surgery, the prevalence of both perceived clinically significant insomnia and cognitive impairment was highest at baseline at 25.84% and lowest at 18 months at 18.73%. We observed strong associations between subjectively assessed insomnia and cognitive functioning, independent of patient age and education. Further, the association between insomnia and cognitive impairments was bidirectional, wherein the presence of one at a single timepoint was significantly associated with the other at the following timepoint. Having comorbid insomnia and cognitive impairment was associated with a few demographic and clinical variables.

In non-cancer samples, there is a large body of experimental evidence to support the insomnia (or sleep deprivation) → perceived cognitive impairment relationship. Sufficient quality and quantity of sleep is essential for optimal attention, concentration, memory, and executive functions (Walker, 2009; Raven et al., 2018). There are several biological mechanisms that have been proposed in an effort to establish the pathways by which sleep disturbances may contribute to cognitive impairment. These include, but are not limited to, changes in neurotransmitter systems involved in sleep (Bekinschtein et al., 2008; Havekes et al., 2012), neural disruptions and reduction in the volume of the hippocampus (Riemann et al., 2007; Meerlo et al., 2009), changes in the functioning of the hypothalamic-pituitary-adrenal (HPA) axis (van Dalfsen and Markus, 2018), and changes in functionality of different brain regions involved in working memory (Mu et al., 2005; Chee et al., 2006). This suggests that an intervention designed to target insomnia may have a secondary benefit of improving perceived cognitive function, but this remains to be tested in cancer survivors (Garland et al., 2021a). Less is known about why perceived or objectively assessed cognitive impairment might be associated with future insomnia, but it is possible that the distress caused by the perception of cognitive impairment (Myers, 2013) might increase insomnia at a subsequent timepoint. Alternatively, there may be some third variable or common etiology (e.g., HPA axis dysregulation) that may explain both (Balbo et al., 2010), but this requires further investigation.

The prevalence of both perceived clinically significant insomnia and cognitive impairment was highest at baseline and declined thereafter, albeit modestly. This is likely due to the acute impact of the distress caused by receiving a cancer diagnosis (Kaiser et al., 2019) or the impacts of a recent surgery (and hospitalization) on sleep (Ida et al., 2019) and cognitions (Wu et al., 2019). The prevalence of clinical levels of perceived cognitive impairment ranged from 37.18 to 39.88% across the 18 months, whereas the prevalence for clinically relevant insomnia symptoms ranged from 35.63 to 55.88%. Our findings further support previous cross-sectional and longitudinal studies demonstrating that roughly 30 percent of breast cancer patients experience perceived cognitive impairment prior to even starting treatment (Wefel et al., 2004; Hermelink et al., 2007). Our results also suggest that perceived cognitive impairment can persist past treatment and remission, in line with past research that up to 35 percent of patients report cognitive impairments from 6 months to 20 years following treatment (Yamada et al., 2010; Koppelmans et al., 2012). We observed that clinical levels of insomnia were more frequent than clinical levels of cognitive impairments, and rates obtained are consistent with past estimates placing the prevalence of cancer-related insomnia to be between 30 and 60%, depending on the type and timing of measurement (Savard and Morin, 2001; Davidson et al., 2002; Savard et al., 2011). The prevalence and relative stability of these two symptoms have important implications for overall health and functioning. Both insomnia and cognitive failures (as measured by the CFQ) have been associated with workplace (Daley et al., 2009; Day et al., 2012) and traffic (Allahyari et al., 2008; Leger et al., 2014) accidents. Research has also demonstrated a direct relationship of insomnia on impaired workplace functioning, including greater absenteeism and presenteeism (Brossoit et al., 2019). Those patients who experience both insomnia and perceived cognitive impairment may be at greater risk for these and other negative impacts on their health and functioning.

A few demographic and clinical variables were significantly associated with the presence of comorbid insomnia and cognitive impairment. Patients with this comorbidity were younger, more likely to be female, and currently undergoing chemotherapy. Previous research has reported associations between younger age and female sex and higher levels of insomnia and perceived cognitive impairment (Oertelt-Prigione et al., 2021). There is also robust evidence to support the association between chemotherapy and both insomnia and perceived cognitive impairment, that persists past treatment completion (Hermelink et al., 2007; Lindner et al., 2014; Janelsins et al., 2017; Yao et al., 2017; Kim et al., 2020). Those who experienced comorbid insomnia and cognitive impairment were also significantly less likely to work than those without clinical levels of insomnia and/or cognitive complaints. This supports the greater combined occupational impacts of having these two conditions. Interestingly, patients with both conditions were less likely to have clinical levels of depression and anxiety, but this can be explained by their greater likelihood of using a psychotropic medication to manage these disorders. None of the other clinical characteristics, including cancer type, or demographic variables were associated with comorbidity.

There are a number of limitations that need to be considered. First, this was a secondary analysis of a larger study designed to assess insomnia over time and not cognitive function. This limited the ability to assess more specific indices of cognitive function. The inclusion of objective measures of cognition would not have been practically feasible with a sample this large. The absence of neuropsychological tests does not allow us to draw conclusions about specific domains of cognitive function associated with insomnia. Further, the measure we used, the CFQ, has a high test-retest reliability which makes it hard to determine whether the relative stability of problems we observed were a result of persistent problems or the measures insensitivity to detect change. Other studies in cancer samples have also not found significant change over time using this measure (Bray et al., 2018). In addition, empirically validated cutoffs for the CFQ have not been established in people diagnosed with cancer. Although patients with an existing diagnosis of a sleep disorder other than insomnia, e.g., sleep apnea, were excluded from this study, those with an undiagnosed sleep disorder may have been undetected. Sleep disorders other than insomnia have also been associated with cognitive complaints. As such, we cannot fully rule out the potential influence of other sleep-related factors in the results observed. Finally, the intervals between time points were fairly long (2 and 4 months) making it more difficult to conclude with certainty about the direction of the relationship between insomnia and cognitive impairment. Additional prospective and clinical studies using both objective and subjective measures of cognitive functioning and shorter time intervals are needed.

Comorbid insomnia and perceived cognitive impairment affects around one in five patients and is more frequent at the beginning of the cancer care trajectory. Reporting these symptoms simultaneously has the potential to exacerbate the overall symptom burden and impact on individual functioning. All patients should be regularly screened for insomnia, cognitive concerns, along with other survivorship issues, followed by a more careful assessment and/or a referral for neuropsychological testing if warranted (Van Dyk and Ganz, 2021). Insomnia may represent an important patient level vulnerability that when identified, can be effectively treated with cognitive-behavioral therapy for insomnia (Johnson et al., 2016), which can improve not only insomnia but also co-occurring fatigue (Heckler et al., 2016), mood disturbance (Peoples et al., 2019), and possibly cognitive impairment.

## Data Availability Statement

The raw data supporting the conclusions of this article will be made available by the authors, without undue reservation.

## Ethics Statement

The studies involving human participants were reviewed and approved by the Research Ethics Committees of the Centre Hospitalier Universitaire de Québec, the Centre Hospitalier Affilié Universitaire de Québec, and the Université Laval. The patients/participants provided their written informed consent to participate in this study.

## Author Contributions

SG: conceptualization, methodology, and writing and editing the original draft. HI: data curation, formal analysis, and editing. JS: funding acquisition, conceptualization, methodology, writing, and editing. All authors contributed to the article and approved the submitted version.

## Conflict of Interest

The authors declare that the research was conducted in the absence of any commercial or financial relationships that could be construed as a potential conflict of interest.

## Publisher’s Note

All claims expressed in this article are solely those of the authors and do not necessarily represent those of their affiliated organizations, or those of the publisher, the editors and the reviewers. Any product that may be evaluated in this article, or claim that may be made by its manufacturer, is not guaranteed or endorsed by the publisher.

## References

[B1] Ahles T. A. Root J. C. (2018). Cognitive Effects of Cancer and Cancer Treatments. *Annu. Rev. Clin. Psychol.* 14 425–451. 29345974 10.1146/annurev-clinpsy-050817-084903 PMC9118140

[B2] Allahyari T. Saraji G. N. Adl J. Hosseini M. Iravani M. Younesian M. (2008). Cognitive failures, driving errors and driving accidents. *Int. J. Occup. Saf. Ergon.* 14 149–158. 10.1080/10803548.2008.11076759 18534151

[B3] American Psychiatric Association (2013). *Diagnostic and statistical manual of mental disorders (DSM-5)*, 5th Edn. Washington, DC: American Psychiatric Association.

[B4] Balbo M. Leproult R. Van Cauter E. (2010). Impact of sleep and its disturbances on hypothalamo-pituitary-adrenal axis activity. *Int. J. Endocrinol.* 2010:759234. 10.1155/2010/759234 20628523 PMC2902103

[B5] Bekinschtein P. Cammarota M. Katche C. Slipczuk L. Rossato J. I. Goldin A. (2008). BDNF is essential to promote persistence of long-term memory storage. *Proc. Natl. Acad. Sci. U S A.* 105 2711–2716. 10.1073/pnas.0711863105 18263738 PMC2268201

[B6] Boscher C. Joly F. Clarisse B. Humbert X. Grellard J. M. Binarelli G. (2020). Perceived Cognitive Impairment in Breast Cancer Survivors and Its Relationships with Psychological Factors. *Cancers* 12:10. 10.3390/cancers12103000 33081111 PMC7602817

[B7] Boyce-van der Wal L. W. Volker W. G. Vliet Vlieland T. P. van den Heuvel D. M. van Exel H. J. Goossens P. H. (2015). Cognitive problems in patients in a cardiac rehabilitation program after an out-of-hospital cardiac arrest. *Resuscitation* 93 63–68. 10.1016/j.resuscitation.2015.05.029 26066808

[B8] Bray V. J. Dhillon H. M. Vardy J. L. (2018). Systematic review of self-reported cognitive function in cancer patients following chemotherapy treatment. *J. Cancer Surviv.* 12 537–559. 10.1007/s11764-018-0692-x 29728959

[B9] Broadbent D. E. Cooper P. F. FitzGerald P. Parkes K. R. (1982). The Cognitive Failures Questionnaire (CFQ) and its correlates. *Br. J. Clin. Psychol.* 21 1–16. 10.1111/j.2044-8260.1982.tb01421.x 7126941

[B10] Brossoit R. M. Crain T. L. Leslie J. J. Hammer L. B. Truxillo D. M. Bodner T. E. (2019). The effects of sleep on workplace cognitive failure and safety. *J. Occup. Health Psychol.* 24 411–422. 10.1037/ocp0000139 30489101 PMC9036851

[B11] Brownlow J. A. Miller K. E. Gehrman P. R. (2020). Insomnia and Cognitive Performance. *Sleep Med. Clin.* 15 71–76. 32005351 10.1016/j.jsmc.2019.10.002 PMC7000136

[B12] Buysse D. J. (2013). Insomnia. *JAMA* 309 706–716. 23423416 10.1001/jama.2013.193 PMC3632369

[B13] Caplette-Gingras A. Savard J. Savard M. H. Ivers H. (2013). Is insomnia associated with cognitive impairments in breast cancer patients? *Behav. Sleep Med.* 11 239–257. 10.1080/15402002.2012.672940 23181706

[B14] Chee M. W. Chuah L. Y. Venkatraman V. Chan W. Y. Philip P. Dinges D. F. (2006). Functional imaging of working memory following normal sleep and after 24 and 35 h of sleep deprivation: Correlations of fronto-parietal activation with performance. *Neuroimage* 31 419–428. 10.1016/j.neuroimage.2005.12.001 16427321

[B15] Daley M. Morin C. M. LeBlanc M. Gregoire J. P. Savard J. Baillargeon L. (2009). Insomnia and its relationship to health-care utilization, work absenteeism, productivity and accidents. *Sleep Med.* 10 427–438. 10.1016/j.sleep.2008.04.005 18753000

[B16] Davidson J. R. MacLean A. W. Brundage M. D. Schulze K. (2002). Sleep disturbance in cancer patients. *Soc. Sci. Med.* 54 1309–1321. 12058848 10.1016/s0277-9536(01)00043-0

[B17] Day A. J. Brasher K. Bridger R. S. (2012). Accident proneness revisited: the role of psychological stress and cognitive failure. *Accid. Anal. Prev.* 49 532–535. 10.1016/j.aap.2012.03.028 23036431

[B18] Fleming L. Randell K. Stewart E. Espie C. A. Morrison D. S. Lawless C. (2018). Insomnia in breast cancer: a prospective observational study. *Sleep* 42:zsy245. 10.1093/sleep/zsy245 30521041

[B19] Garland S. N. Savard J. Dalton K. Walsh N. A. Seal M. Rash J. (2021a). Rationale and protocol for a randomized waitlist controlled trial of videoconference delivered cognitive behaviour therapy for insomnia (CBT-I) to improve perceived cognitive impairment (PCI) among cancer survivors. *Contemp. Clin. Trials* 103:106322. 10.1016/j.cct.2021.106322 33588077

[B20] Garland S. N. Savard J. Eisel S. L. Wassersug R. J. Rockwood N. J. Thoms J. (2021b). A 2-year prospective analysis of insomnia as a mediator of the relationship between androgen deprivation therapy and perceived cognitive function in men with prostate cancer. *Cancer* 127 4656–4664. 10.1002/cncr.33850 34411294 PMC8664983

[B21] Goel N. Rao H. Durmer J. S. Dinges D. F. (2009). Neurocognitive consequences of sleep deprivation. *Semin. Neurol.* 29 320–339. 10.1055/s-0029-1237117 19742409 PMC3564638

[B22] Harrold E. C. Idris A. F. Keegan N. M. Corrigan L. Teo M. Y. O’Donnell M. (2020). Prevalence of Insomnia in an Oncology Patient Population: An Irish Tertiary Referral Center Experience. *J. Natl. Compr. Canc. Netw.* 18 1623–1630. 10.6004/jnccn.2020.7611 33285516 PMC9337980

[B23] Havekes R. Vecsey C. G. Abel T. (2012). The impact of sleep deprivation on neuronal and glial signaling pathways important for memory and synaptic plasticity. *Cell Signal.* 24 1251–1260. 10.1016/j.cellsig.2012.02.010 22570866 PMC3622220

[B24] Heckler C. E. Garland S. N. Peoples A. R. Perlis M. L. Shayne M. Morrow G. R. (2016). Cognitive behavioral therapy for insomnia, but not armodafinil, improves fatigue in cancer survivors with insomnia: a randomized placebo-controlled trial. *Support Care Cancer* 24 2059–2066. 10.1007/s00520-015-2996-y 26542272 PMC4805518

[B25] Hermelink K. Untch M. Lux M. P. Kreienberg R. Beck T. Bauerfeind I. (2007). Cognitive function during neoadjuvant chemotherapy for breast cancer: results of a prospective, multicenter, longitudinal study. *Cancer* 109 1905–1913. 10.1002/cncr.22610 17351951

[B26] Hoang H. T. X. Molassiotis A. Chan C. W. Nguyen T. H. Liep Nguyen V. (2019). New-onset insomnia among cancer patients undergoing chemotherapy: prevalence, risk factors, and its correlation with other symptoms. *Sleep Breath* 24 241–251. 10.1007/s11325-019-01839-x 31016572

[B27] Ida M. Onodera H. Yamauchi M. Kawaguchi M. (2019). Preoperative sleep disruption and postoperative functional disability in lung surgery patients: a prospective observational study. *J. Anesth.* 33 501–508. 10.1007/s00540-019-02656-y 31190287

[B28] Innominato P. F. Spiegel D. Ulusakarya A. Giacchetti S. Bjarnason G. A. Levi F. (2015). Subjective sleep and overall survival in chemotherapy-naive patients with metastatic colorectal cancer. *Sleep Med.* 16 391–398. 10.1016/j.sleep.2014.10.022 25678361

[B29] Janelsins M. C. Heckler C. E. Peppone L. J. Ahles T. A. Mohile S. G. Mustian K. M. (2018). Longitudinal Trajectory and Characterization of Cancer-Related Cognitive Impairment in a Nationwide Cohort Study. *J. Clin. Oncol.* 2018:JCO2018786624. 10.1200/JCO.2018.78.6624 30240328 PMC6225503

[B30] Janelsins M. C. Heckler C. E. Peppone L. J. Kamen C. Mustian K. M. Mohile S. G. (2017). Cognitive Complaints in Survivors of Breast Cancer After Chemotherapy Compared With Age-Matched Controls: An Analysis From a Nationwide, Multicenter, Prospective Longitudinal Study. *J. Clin. Oncol.* 35 506–514. 10.1200/JCO.2016.68.5826 28029304 PMC5455314

[B31] Johnson J. A. Rash J. A. Campbell T. S. Savard J. Gehrman P. R. Perlis M. (2016). A systematic review and meta-analysis of randomized controlled trials of cognitive behavior therapy for insomnia (CBT-I) in cancer survivors. *Sleep Med. Rev.* 27 20–28. 10.1016/j.smrv.2015.07.001 26434673

[B32] Kaiser J. Dietrich J. Amiri M. Ruschel I. Akbaba H. Hantke N. (2019). Cognitive Performance and Psychological Distress in Breast Cancer Patients at Disease Onset. *Front Psychol.* 10:2584. 10.3389/fpsyg.2019.02584 31803117 PMC6873390

[B33] Kim H. J. Jung S. O. Kim H. Abraham I. (2020). Systematic review of longitudinal studies on chemotherapy-associated subjective cognitive impairment in cancer patients. *Psychooncology* 29 617–631. 10.1002/pon.5339 32017297

[B34] Koppelmans V. Breteler M. M. Boogerd W. Seynaeve C. Gundy C. Schagen S. B. (2012). Neuropsychological performance in survivors of breast cancer more than 20 years after adjuvant chemotherapy. *J. Clin. Oncol.* 30:1080. 10.1200/JCO.2011.37.0189 22370315

[B35] Lange M. Joly F. Vardy J. Ahles T. Dubois M. Tron L. (2019). Cancer-related cognitive impairment: an update on state of the art, detection, and management strategies in cancer survivors. *Ann. Oncol.* 30 1925–1940. 10.1093/annonc/mdz410 31617564 PMC8109411

[B36] Leger D. Bayon V. Ohayon M. M. Philip P. Ement P. Metlaine A. (2014). Insomnia and accidents: cross-sectional study (EQUINOX) on sleep-related home, work and car accidents in 5293 subjects with insomnia from 10 countries. *J. Sleep Res.* 23 143–152. 10.1111/jsr.12104 24237855

[B37] Leysen L. Lahousse A. Nijs J. Adriaenssens N. Mairesse O. Ivakhnov S. (2019). Prevalence and risk factors of sleep disturbances in breast cancersurvivors: systematic review and meta-analyses. *Support Care Cancer* 27 4401–4433. 10.1007/s00520-019-04936-5 31346744

[B38] Lindner O. C. Phillips B. McCabe M. G. Mayes A. Wearden A. Varese F. (2014). A meta-analysis of cognitive impairment following adult cancer chemotherapy. *Neuropsychology* 28 726–740. 10.1037/neu0000064 24635712 PMC4143183

[B39] Lowery-Allison A. E. Passik S. D. Cribbet M. R. Reinsel R. A. O’Sullivan B. Norton L. (2018). Sleep problems in breast cancer survivors 1-10 years posttreatment. *Palliat. Support Care.* 16 325–334. 10.1017/S1478951517000311 28508735

[B40] Maguire R. Drummond F. J. Hanly P. Gavin A. Sharp L. (2019). Problems sleeping with prostate cancer: exploring possible risk factors for sleep disturbance in a population-based sample of survivors. *Support Care Cancer* 27 3365–3373. 10.1007/s00520-018-4633-z 30627919

[B41] Meerlo P. Mistlberger R. E. Jacobs B. L. Heller H. C. McGinty D. (2009). New neurons in the adult brain: the role of sleep and consequences of sleep loss. *Sleep Med. Rev.* 13 187–194. 10.1016/j.smrv.2008.07.004 18848476 PMC2771197

[B42] Moulaert V. R. M. van Heugten C. M. Gorgels T. P. M. Wade D. T. Verbunt J. A. (2017). Long-term Outcome After Survival of a Cardiac Arrest: A Prospective Longitudinal Cohort Study. *Neurorehabil. Neural. Repair.* 31 530–539. 10.1177/1545968317697032 28506147

[B43] Mu Q. Nahas Z. Johnson K. A. Yamanaka K. Mishory A. Koola J. (2005). Decreased cortical response to verbal working memory following sleep deprivation. *Sleep* 28 55–67. 10.1093/sleep/28.1.55 15700721

[B44] Myers J. S. (2013). Cancer- and chemotherapy-related cognitive changes: the patient experience. *Semin Oncol. Nurs.* 29 300–307. 10.1016/j.soncn.2013.08.010 24183161

[B45] Nishiura M. Tamura A. Nagai H. Matsushima E. (2015). Assessment of sleep disturbance in lung cancer patients: relationship between sleep disturbance and pain, fatigue, quality of life, and psychological distress. *Palliat. Support Care.* 13 575–581. 10.1017/S1478951513001119 24524428

[B46] Oertelt-Prigione S. de Rooij B. H. Mols F. Oerlemans S. Husson O. Schoormans D. (2021). Sex-differences in symptoms and functioning in >5000 cancer survivors: Results from the PROFILES registry. *Eur. J. Cancer.* 156 24–34. 10.1016/j.ejca.2021.07.019 34411849

[B47] Ohayon M. M. (2002). Epidemiology of insomnia: what we know and what we still need to learn. *Sleep Med. Rev.* 6 97–111. 10.1053/smrv.2002.0186 12531146

[B48] Palesh O. G. Roscoe J. A. Mustian K. M. Roth T. Savard J. Ancoli-Israel S. (2010). Prevalence, demographics, and psychological associations of sleep disruption in patients with cancer: University of Rochester Cancer Center-Community Clinical Oncology Program. *J. Clin. Oncol.* 28 292–298. 10.1200/JCO.2009.22.5011 19933917 PMC2815717

[B49] Peoples A. R. Garland S. N. Pigeon W. R. Perlis M. L. Wolf J. R. Heffner K. L. (2019). Cognitive Behavioral Therapy for Insomnia Reduces Depression in Cancer Survivors. *J. Clin. Sleep Med.* 15 129–137. 10.5664/jcsm.7586 30621831 PMC6329536

[B50] Raven F. Van der Zee E. A. Meerlo P. Havekes R. (2018). The role of sleep in regulating structural plasticity and synaptic strength: Implications for memory and cognitive function. *Sleep Med. Rev.* 39 3–11. 10.1016/j.smrv.2017.05.002 28641933

[B51] Riemann D. Voderholzer U. Spiegelhalder K. Hornyak M. Buysse D. J. Nissen C. (2007). Chronic insomnia and MRI-measured hippocampal volumes: a pilot study. *Sleep* 30 955–958. 10.1093/sleep/30.8.955 17702263 PMC1978381

[B52] Rodriguez N. Fawcett J. M. Rash J. A. Lester R. Powell E. MacMillan C. D. (2021). Factors associated with cognitive impairment during the first year of treatment for nonmetastatic breast cancer. *Cancer Med.* 10 1191–1200. 10.1002/cam4.3715 33455070 PMC7926005

[B53] Ruel S. Savard J. Ivers H. (2015). Insomnia and self-reported infections in cancer patients: An 18-month longitudinal study. *Health Psychol.* 34 983–991. 10.1037/hea0000181 25603417

[B54] Savard J. Ivers H. Villa J. Caplette-Gingras A. Morin C. M. (2011). Natural course of insomnia comorbid with cancer: an 18-month longitudinal study. *J. Clin. Oncol.* 29 3580–3586. 10.1200/JCO.2010.33.2247 21825267

[B55] Savard J. Morin C. M. (2001). Insomnia in the context of cancer: a review of a neglected problem. *J. Clin. Oncol.* 19 895–908. 10.1200/JCO.2001.19.3.895 11157043

[B56] Savard J. Villa J. Ivers H. Simard S. Morin C. M. (2009). Prevalence, natural course, and risk factors of insomnia comorbid with cancer over a 2-month period. *J. Clin. Oncol.* 27 5233–5239. 10.1200/JCO.2008.21.6333 19738124

[B57] Savard M. H. Savard J. Simard S. Ivers H. (2005). Empirical validation of the Insomnia Severity Index in cancer patients. *Psychooncology* 14 429–441. 10.1002/pon.860 15376284

[B58] Steinbusch C. V. M. van Heugten C. M. Rasquin S. M. C. Verbunt J. A. Moulaert V. R. M. (2017). Cognitive impairments and subjective cognitive complaints after survival of cardiac arrest: A prospective longitudinal cohort study. *Resuscitation* 120 132–137. 10.1016/j.resuscitation.2017.08.007 28818523

[B59] van Dalfsen J. H. Markus C. R. (2018). The influence of sleep on human hypothalamic-pituitary-adrenal (HPA) axis reactivity: A systematic review. *Sleep Med. Rev.* 39 187–194. 10.1016/j.smrv.2017.10.002 29126903

[B60] Van Dyk K. Ganz P. A. (2021). Cancer-Related Cognitive Impairment in Patients With a History of Breast Cancer. *JAMA* 326 1736–1737. 10.1001/jama.2021.13309 34652424

[B61] Walker M. P. (2009). The role of sleep in cognition and emotion. *Ann. N. Y. Acad. Sci.* 1156 168–197. 10.1111/j.1749-6632.2009.04416.x 19338508

[B62] Wefel J. S. Lenzi R. Theriault R. L. Davis R. N. Meyers C. A. (2004). The cognitive sequelae of standard-dose adjuvant chemotherapy in women with breast carcinoma: results of a prospective, randomized, longitudinal trial. *Cancer* 100 2292–2299. 15160331 10.1002/cncr.20272

[B63] Wu L. Zhao H. Weng H. Ma D. (2019). Lasting effects of general anesthetics on the brain in the young and elderly: “mixed picture” of neurotoxicity, neuroprotection and cognitive impairment. *J. Anesth.* 33 321–335. 10.1007/s00540-019-02623-7 30859366 PMC6443620

[B64] Yamada T. H. Denburg N. L. Beglinger L. J. Schultz S. K. (2010). Neuropsychological outcomes of older breast cancer survivors: cognitive features ten or more years after chemotherapy. *J. Neuropsychiatry Clin. Neurosci.* 22:48. 10.1176/jnp.2010.22.1.48 20160209 PMC3641161

[B65] Yao C. Bernstein L. J. Rich J. B. (2017). Executive functioning impairment in women treated with chemotherapy for breast cancer: a systematic review. *Breast Cancer Res. Treat.* 166:15. 10.1007/s10549-017-4376-4 28707202

